# AMD Genomics: Non-Coding RNAs as Biomarkers and Therapeutic Targets

**DOI:** 10.3390/jcm11061484

**Published:** 2022-03-09

**Authors:** Charles Zhang, Leah A. Owen, John H. Lillvis, Sarah X. Zhang, Ivana K. Kim, Margaret M. DeAngelis

**Affiliations:** 1 Department of Ophthalmology, Ross Eye Institute, Jacobs School of Medicine and Biomedical Sciences, State University of New York, University at Buffalo, Buffalo, NY 14203, USA; czhang62@buffalo.edu (C.Z.); leah.owen@hsc.utah.edu (L.A.O.); jhlillvi@buffalo.edu (J.H.L.); xzhang38@buffalo.edu (S.X.Z.); 2 Department of Ophthalmology and Visual Sciences, University of Utah School of Medicine, The University of Utah, Salt Lake City, UT 84132, USA; 3 Department of Population Health Sciences, University of Utah School of Medicine, The University of Utah, Salt Lake City, UT 84132, USA; 4 Department of Obstetrics and Gynecology, University of Utah School of Medicine, The University of Utah, Salt Lake City, UT 84132, USA; 5 Veterans Administration Western New York Healthcare System, Buffalo, NY 14212, USA; 6 Department of Biochemistry, Jacobs School of Medicine and Biomedical Sciences, State University of New York, University at Buffalo, Buffalo, NY 14203, USA; 7 Neuroscience Graduate Program, Jacobs School of Medicine and Biomedical Sciences, State University of New York, University at Buffalo, Buffalo, NY 14203, USA; 8 Retina Service, Massachusetts Eye & Ear, Department of Ophthalmology, Harvard Medical School, Boston, MA 02114, USA; 9 Genetics, Genomics and Bioinformatics Graduate Program, Jacobs School of Medicine and Biomedical Sciences, State University of New York, University at Buffalo, Buffalo, NY 14203, USA

**Keywords:** age-related macular degeneration, genomics, non-coding RNAs, biomarkers, therapeutics

## Abstract

Age-related macular degeneration (AMD) is a progressive neurodegenerative disease that is the world’s leading cause of blindness in the aging population. Although the clinical stages and forms of AMD have been elucidated, more specific prognostic tools are required to determine when patients with early and intermediate AMD will progress into the advanced stages of AMD. Another challenge in the field has been the appropriate development of therapies for intermediate AMD and advanced atrophic AMD. After numerous negative clinical trials, an anti-C5 agent and anti-C3 agent have recently shown promising results in phase 3 clinical trials, in terms of slowing the growth of geographic atrophy, an advanced form of AMD. Interestingly, both drugs appear to be associated with an increased incidence of wet AMD, another advanced form of the disease, and will require frequent intravitreal injections. Certainly, there remains a need for other therapeutic agents with the potential to prevent progression to advanced stages of the disease. Investigation of the role and clinical utility of non-coding RNAs (ncRNAs) is a major advancement in biology that has only been minimally applied to AMD. In the following review, we discuss the clinical relevance of ncRNAs in AMD as both biomarkers and therapeutic targets.

## 1. Background

Age-related macular degeneration (AMD) is a progressive neurodegenerative disease that is the world’s leading cause of blindness in the aging population [1]. Approximately 11 million individuals are affected by AMD in the United States (US), with a global prevalence of 170 million. In the US, the prevalence is similar to all invasive cancers combined and more than double that of Alzheimer’s disease [1,2,3]. It is estimated that the direct healthcare costs from AMD amount to $4.6 billion each year in the US [4]. Due to the increased prevalence with age and changing demographics of the US population, it is anticipated that the number of US patients with AMD will reach 22 million by the year 2050, with the expenditures to increase proportionally [5].

Drusen are the hallmark of the disease and appear as yellowish lipid-rich, protein-containing deposits that accumulate between the retinal pigment epithelium (RPE) and Bruch’s membrane. As the burden of these deposits increases, the disease can be categorized into early, intermediate, or advanced stages, also taking into account hyper- or hypo-pigmentary changes of the RPE and the presence or absence of macular neovascularization (MNV) and areas of atrophy of the RPE and outer retina [6]. 

Commonly utilized classifications systems include the Age-Related Eye Disease Study (AREDS) grading scale which includes four categories of non-advanced disease [6,7]. The more recent and further simplified Beckman classification categorizes eyes into those with normal aging changes and early, intermediate, and late AMD [8]. The tools used to assist in staging include fundus imaging of the retina for direct visualization of drusen deposits, pigmentary changes of the RPE, and exudative changes in the retina [9,10,11], intravenous fluorescein angiography to aid in detecting MNV, and spectral domain optical coherence tomography (SD-OCT) to provide high-resolution, non-invasive cross-sectional, en-face, and angiographic imaging of the retina [9,12]. 

Advanced forms of AMD manifest in both a non-neovascular or dry form and a neovascular or wet form. The clinical presentation of these two differ dramatically, in that the wet form can cause rapid severe vision loss secondary to the development of new abnormal blood vessels in the normally avascular sub-RPE and sub-retinal regions, previously termed choroidal neovascularization, more recently named macular neovascularization [13]. Wet AMD represents a small proportion of total AMD cases and development of anti-vascular endothelial growth factor (VEGF) treatment has made tremendous strides in reducing the visual loss associated with the disease [14]. However, despite the ability of the medications to often reverse acute vision loss through VEGF inhibition, there is persistent progression of visual deterioration despite control of the neovascularization [15,16].

The clinical course towards advanced dry AMD is typically gradual and accompanied by an increasing burden of drusen, RPE pigmentary abnormalities, and subretinal deposits known as reticular pseudodrusen. In the early phases, central visual acuity is relatively well maintained and the symptoms, if present, typically consist of decreased contrast sensitivity and poor dark adaptation [17]. The gradual accumulation of the drusen ultimately leads to loss of the RPE and overlying photoreceptors that, when confluent, are known as the clinical entity of geographic atrophy (GA) [18]. Once GA is present, patients tend to inevitably progress to foveal involvement, which leads to debilitating central visual acuity degradation [19,20]. Consequently, AMD patients continue to represent a large population of individuals referred for low vision rehabilitation due to irreversible vision loss.

### 1.1. Current Challenges in AMD

Although the clinical stages and forms of AMD have been elucidated, more specific prognostic tools are required to determine when patients with early and intermediate AMD will progress into the advanced stages of AMD, and into which form of advanced disease. Longitudinal studies with large cohorts have identified signs associated with risk of progression. Studies have highlighted the appearance of precursor lesions to GA [21], as well as measured the rate of progression of GA once it occurs [19,20,22]. Recent work has incorporated artificial intelligence to predict risk of conversion to wet AMD [23]. 

Another major challenge in the field has been the development of therapies for intermediate AMD and advanced atrophic AMD. To date, the only approved treatment for intermediate AMD is supplementation of antioxidant micronutrients (AREDS2 formula), which has been demonstrated only to modestly reduce the rate of progression to advanced AMD [6]. Studies investigating the underlying pathophysiologic processes behind GA have suggested multiple pathways including oxidative stress, lipid dysregulation, inflammation, and complement that could be targeted with traditional small molecules and antibody treatments [24,25,26,27,28]. After numerous negative clinical trials, an anti-C5 agent and anti-C3 agent have recently shown promising results in phase 3 clinical trials in terms of slowing the growth of geographic atrophy [29,30]. However, it remains to be seen whether FDA approval will follow. Interestingly, both drugs appear to be associated with an increased incidence of wet AMD and will require frequent intravitreal injections. Certainly, there remains a need for other therapeutic agents with the potential to prevent progression to this advanced stage of disease.

### 1.2. The Role of ncRNAs in AMD

Investigation of the role and clinical utility of non-coding RNAs (ncRNAs) is a major advancement in biology that has only been minimally applied to AMD [31,32,33,34,35,36]. The term non-coding RNA generally is used to describe RNA that does not encode a protein. However, just because an RNA does not translate into a protein does not mean that it does not have an important biological function. Approximately 99% of the transcriptionally active human genome does not encode proteins, but rather gives rise to a broad spectrum of ncRNAs with important regulatory and structural functions. When comparing the dramatic increase in the number of ncRNAs and the modest increase in protein-coding genes between simple organisms and humans, it is theorized that ncRNAs are vital in primate cellular physiology. Regarding disease processes, the Encyclopedia of DNA Elements (ENCODE) project and other studies have demonstrated that non-coding variants in the genome play an important role in the pathogenesis of many complex disorders, including AMD, and represent a relatively untapped pool of potential biomarkers of underlying disease processes [37,38,39,40,41,42,43].

These ncRNAs differ from coding RNA or messenger ribonucleic acid (mRNA) in that they do not encode for proteins, but rather influence gene expression through a variety of means. Some of the more well-studied ncRNAs include small interfering RNAs (siRNAs), and micro RNAs (miRNAs or miRs) have been shown to play a role in the inhibition of gene expression through RNA interference (RNAi) [44]. Others such as long noncoding RNAs (lncRNAs), circular RNAs (circRNA), and piwi interacting RNAs (piRNAs) have less well described mechanisms of manipulating gene expression that are only beginning to be uncovered [45,46]. Of these ncRNAs, miRs are probably the most studied in AMD (Table 1) [38,42,47,48,49,50,51,52,53,54,55,56]. Briefly miRs, or short ncRNAs, are transcribed by Polymerase II a, capped, poly-adenylated, and spliced similar to coding RNAs or mRNAs. Compared to mRNAs, miRs (20–24 nucleotides in length) influence gene expression by binding imperfectly to a target mRNA(s), generally at the 3′ end and can repress or activate translation. In this manner, the miRs can control the flow of genetic information. For a more in-depth understanding of miRs and their putative roles in human disease in general, please see the excellent reviews [57,58,59]. 

Several studies have investigated miRs as potential biomarkers for AMD and in turn hypothesized that these small ncRNAs may be therapeutic targets. These studies obtained human tissue samples from a variety of sources including vitreous, peripheral blood cells, plasma, and serum of patients with late AMD, either exudative or neovascular disease, or patients with GA. In these studies, ncRNAs are primarily interrogated via a candidate miR approach (as opposed to an agnostic approach to examine the entire miR genome, with directmiR sequencing) to quantify miR expression differences between AMD patients and controls. The results between studies are inconsistent, with a handful of miRs overlapping between studies as potential biomarkers of AMD. Based on consistency among studies of the 53 identified markers (Table 1), 7 (miR-17, -23a, -27a, -126, -146a, -155, and -410) were found to be significantly associated with AMD. For example, while miR-17 was found between two studies to be associated with AMD, mir-17 was found to have lower expression in wet AMD in one study [53] while having higher expression wet AMD in another [50], depending on the tissue being examined. 

The most consistent association between studies involves miR-126, as it was elevated in peripheral blood and serum in patients with wet AMD by more than one study [42,52,53]. miR-126 is postulated to play a role in regulating angiogenesis in endothelial cells [60,61]. Knockout mouse studies have suggested bioinformatically that the proangiogenic role of miR-126 within the vascular endothelium is through promotion of VEGF signaling by negative regulation of Sprouty-related EVH domain containing protein (SPRED1) and PI3K regulatory subunit 2 (PIK3R2), which are negative regulators of the RAF and AKT signaling pathways downstream of VEGF, respectively [62,63,64]. Again, utilizing a bioinformatic approach, a purported mechanism of miR-126 includes downregulation of calmodulin regulated spectrin associated protein 1, which results in promotion of microtubule arrangement of the sprouting of angiogenesis [65,66]. Other possible targets of miR-126 are the protein tyrosine phosphatase, non-receptor type 9, which is associated with the promotion of cellular proliferation and angiogenesis. However, the exact mechanism of regulation of this protein is unclear [67,68]. 

miR-146a could also be a promising biomarker as it was similarly elevated in three studies evaluating AMD patients versus normal controls [49,52,53]. miR-146a has many gene targets and has been associated with other neurodegenerative disorders including Alzheimer’s disease [69]. Studies have demonstrated that miR-146a may dampen innate immunity through interference of the Interleukin 1 receptor associated kinase 1 (IRAK1) and TNF receptor associated factor 6 (TRAF-6), as well as down-regulation of IL-6 [70,71,72]. Inflammation has been associated with AMD as a whole; IL-1 and IL-6 have been found to be associated with MNV [71,73]. Additionally, studies have suggested that mi-146a may negatively regulate complement factor H in both neural and endothelial tissue [74,75,76]. These findings remain to be validated in human RPE and/or neural retina.

Conflicting reports on expression levels of miR-17 (Table 1) could potentially be explained in that one study evaluated extracellular levels in plasma [50], while the other investigated peripheral blood cells [53]. miRNA findings between studies may be inconsistent for a variety of reasons, including differences in tissue from which miR is obtained, variation in phenotyping of samples, heterogeneity in phenotype, ascertainment and collection techniques, and differences in ethnicities of the populations. Future studies aimed at investigating the roles of miRNAs in AMD will likely require more stringent control of these variables and should follow the suggested design paradigms highlighted in the discussion below.

An overall important consideration in study design is that similar to RNA, non-coding RNAs are tissue and cell specific [24,77,78,79,80,81,82]. Given that it is still unknown if pathogenic changes in AMD are localized to specific ocular tissues or systemic, one must take into consideration that potential biomarkers identified in the peripheral blood as “disease associated” may not reflect the disease mechanism occurring in the neural retina and/or RPE. 

In the following review, we discuss the clinical relevance of ncRNAs in AMD. The first major goal is to illustrate the role ncRNAs may play in serving as potential biomarkers to help supplement our current image-based diagnostic paradigm, as well as illustrate their role as prognostic tools. The second goal is to hypothesize the potential role that ncRNA may play as therapeutic targets to expand our repertoire of clinical agents to manage this debilitating condition. 

## 2. ncRNAs as Potential Biomarkers

### 2.1. Targets for Using ncRNAs as Biomarkers

Our current method of diagnosing AMD relies on imaging findings of drusen, GA, or MNV that are reflective of currently unclear processes occurring at the cellular level. Though we have an incomplete understanding of what exactly causes these changes to occur, many studies investigating the pathophysiologic pathways activated in AMD shed light into potential ncRNA biomarkers. 

Given the high metabolic activity and continuous light exposure, the macula is an area of high oxidative burden which is believed to be involved in drusenogenesis [83]. The importance of this pathway is supported by studies demonstrating that high oxidative stress may be the result of lifestyle choices, such as cigarette smoking and high fat and high glycemic index diets, are associated with an increased risk of developing AMD [24,83,84,85,86]. Furthermore, genetic variants in oxidative stress genes, such as MTND2*LHON-4917G, NADH subunits, SOD2, and PPARGC1A, have been associated with an increased risk of AMD [87,88,89]. In cardiovascular medicine, multiple miRs associated with oxidative stress have been found to be upregulated after myocardial infarctions [90,91]. Some of these non-coding molecules have been proposed to be used as biomarkers for early diagnosis of myocardial infarction and heart failure with the added advantage of earlier detection than traditional biomarkers such as troponin or CKMB [92]. Moreover, these data have been refined into a unique 20-miR signature obtained from whole blood that could predict acute myocardial infarction with a higher specificity and sensitivity than troponin [93,94].

There has been growing evidence that dysregulation of lipids and lipoproteins plays a role in AMD. Lipids not only compose 40% of drusen by volume, but there is evidence that accumulation of cholesterol from RPE phagocytosis of photoreceptor outer segments or from ingestion of lipoproteins from the circulation play a critical role in drusenogenesis [95,96,97]. Furthermore, several studies have identified lipoprotein-related genes, such as LIPC, CETP, ACBA1, and APOE, that have been associated with an increased risk of AMD [98,99,100,101]. Recent metabolomic investigations have also confirmed the role of lipid pathways in AMD pathogenesis [102,103]. Studies in cardiovascular medicine have sought to identify ncRNA molecules, whose expression was changed in coronary artery disease atherogenesis and identified multiple lncRNAs and miRNAs that may serve as potential biomarkers for cardiovascular disease [104,105,106,107].

Perhaps the most studied pathway of AMD pathogenesis lies in the dysregulation of the immune and complement system. Early studies have found that polymorphisms in complement factor H, which accounts for a major component of the heritability of AMD, has led to the identification of multiple genes associated with increased AMD risk, though our understanding is incomplete in how these abnormalities directly lead to AMD [98,106,107,108,109,110,111,112,113]. Studies have also demonstrated that immune and complement dysregulation in AMD patients is not limited to the RPE, but also occurs systemically as demonstrated by alterations of inflammatory cytokine profiles [114,115,116,117]. Studies have demonstrated alterations in miRNAs that control inflammation in both retinal tissue and blood plasma in patients with AMD compared to controls [41,118].

### 2.2. Clinical Benefit of Using ncRNAs as Biomarkers

Our current methodology of diagnosing AMD has advanced tremendously since the original AREDS criteria and now can include imaging modalities in addition to color fundus photography, including fundus autofluorescence (FAF), OCT, and most recently OCT angiography (OCTA). Additionally, recent studies have attempted to utilize artificial intelligence on imaging data, working towards using these advanced imaging technologies to help prognosticate disease progression. However, the major limitation is that all these imaging changes represent major geographic changes of the RPE and photoreceptor complex, rather than the underlying cellular pathophysiologic processes themselves. One of the unique advantages of using ncRNA as biomarkers is that they are representative of the intracellular processes. This may provide the benefit of earlier diagnosis and provide additional prognostic information, as well as play a role in biomarker-guided therapy for further personalization of AMD treatment.

Though some studies have demonstrated that a loss of confluence of drusen is the typical pattern that precedes the onset of geographic atrophy, accurate prediction of GA prior to its appearance remains difficult [21]. Multiple other medical fields have demonstrated that ncRNA may provide earlier diagnosis of specific disease processes both acute and chronic. As mentioned above, miRNA signatures have been evaluated as potential biomarkers for early diagnosis of myocardial infarction prior to the onset of typical markers such as CKMB or troponin [93,94]. In Alzheimer’s disease, a condition for which confirmatory diagnosis typically requires postmortem phenotyping, a 7-signature miR could be used to predict disease with high accuracy [119]. This has been even further validated using a hybrid of miRNA and piRNA signatures along with traditional protein markers to generate an AUC of 0.98, while also demonstrating the utility of piRNA as biomarkers for disease [120].

In regard to using ncRNA in disease prognostication, studies in cardiology have demonstrated that ncRNA has the potential to predict future cardiac risk with higher fidelity than traditional models. A lncRNA, LIPCAR has been shown to predict cardiovascular mortality in heart failure patients and can be used in conjunction with traditional biomarkers [121]. Additionally, ANRIL and KCNQ1OT1 have been used in combination with existing models to predict left ventricular dysfunction in the prognostication of post-MI patients [122]. 

Finally, a theoretical benefit of using ncRNAs, including miRs, as biomarkers lies in their potential role in serving as an instrumental role in biomarker-guided therapy. As seen in those with neovascular variants of AMD, the interindividual variability in the response to anti-VEGF treatment is a difficult topic for clinicians to address. Some individuals respond readily to a short course of injections and regress without further treatment, whereas others require continued treatment for extended and often indefinite periods of time. While there are many studies aiming to identify risk factors, current therapy is very difficult to tailor in a data-driven manner. Tailored therapies based on an understanding of the underlying molecular characteristics could be administered to those who are anticipated to benefit most, while simultaneously limiting ineffective or harmful interventions. The cardiovascular literature has suggested that ncRNAs have the potential to guide antiplatelet therapy [123,124,125,126,127,128,129], cardiac resynchronization therapy [130,131], left ventricular assist device therapy [132,133,134], and antihypertensive therapy [135]. Given that cardiovascular disease has some overlapping risk factors with AMD pathogenesis (e.g., hypertension, age, family history, and hyperlipidemia), one might hypothesize that similar approaches may demonstrate the utility of ncRNAs as biomarkers in augmenting future AMD therapies. 

Furthermore, it is possible that there could be more sensitive signals of atrophy progression than the current standard of fundus autofluorescence imaging. FAF is still susceptible to limitations, including media opacities and blue light absorbing pigment in the macula [136]. Additionally, studies have demonstrated that atrophy areas measured by FAF and SD-OCT are inconsistent and may be unreliable as therapeutic endpoints [137]. We hypothesize that it may be possible that certain ncRNAs may augment imaging as biomarkers of GA burden, which could potentially be used as additional endpoints in clinical trials.

### 2.3. Challenges of ncRNAs as Biomarkers

Pilot studies on genome-wide associations, comprehensive sequencing work, and animal models of AMD have been instrumental in identifying much of our current understanding of the pathophysiology of AMD [98]. However, due to their intrinsic differences between species, ncRNAs may be difficult to investigate using the traditional research paradigm of cell to animal to human [138]. The function of the same ncRNA may differ drastically between species, and many animal models may differ in their mechanism of disease from their human homologue. Most importantly, some ncRNAs present in murine models do not exist within humans, and therefore their potential role as a potential biomarker in humans will translate poorly [139]. Moreover, proof-of-concept studies in traditional animal models may not be feasible. However, lack of such study should not be a reason to dissuade further research in the subject. 

Another challenge we face with using ncRNAs as biomarkers is that they are predominantly found intracellularly [140,141,142,143]. Therefore, deciding on an appropriate biologic source for a biomarker is vital in designing appropriate translational studies. Changes in ncRNA will help to elucidate the cellular pathways that are dysregulated in AMD, and in order to precisely detect these changes in ncRNA, it will be important to directly sample well-phenotyped RPE and photoreceptor cells [24,81,82,144]. A more pragmatic approach is likely one that relies on serum and plasma ncRNA, which have been demonstrated to correlate with ocular processes such as neuroprotection and angiogenesis [145]. Such an approach has been examined in AMD-related studies related to inflammation and lipogenesis in the choroidal vasculature [146,147]. However, it remains to be seen whether changes in RPE and neural retina physiology can be detected with peripheral serum and plasma samples. Unique to ophthalmology, the aqueous humor has been investigated in some studies as a source for ncRNA biomarkers, whereby lncRNAs have been proposed as biomarkers in primary open angle glaucoma [148]. In the case of AMD, the vitreous may be a biological surrogate to study the RPE and/or neural retina.

Finally, the more practical constraint imposed by using ncRNAs is that these molecules are inherently ephemeral molecules with limited long-term stability [149,150,151,152]. High fidelity and rigorous standardization are necessary to ensure appropriate targets that would be widely accepted and applied in clinical practice to provide their purported benefits. Of the ncRNAs, most studies have focused on miRs due to their stability in circulation, resistance to RNAse digestion, and ability to remain intact despite extreme pH, high temperature, extended storage, and multiple freeze-thaw cycles [153]. However, despite these benefits, studies evaluating cutoffs for diagnostic purposes must account for variations in transportation decay, freeze thawing, and the lab-to-lab inconsistency of extracting RNA from blood and serum when determining cutoff points. Additionally, although the cost of sequencing has improved tremendously over the past 30 years, it continues to be a costly endeavor when considering the population that requires testing [154,155,156]. Although AMD is an enormous financial burden to society, in an economically constrained world it would be important to selectively redirect resources to diagnostics with the greatest cost to benefit ratio.

### 2.4. Guidance in Using ncRNAs as Biomarkers

To address these challenges, we propose several important pillars to follow to improve the yield of future studies. One of the benefits of the high throughput RNA sequence technology and full transcriptome sequencing is that it allows for the assessment of many ncRNAs molecules simultaneously. However, it is vital that any molecules chosen for investigation should be ones with a known biologic function in vivo and ones where a plausible hypothesis of their up or downregulation is generated. It should be emphasized that for these ncRNA to be considered as potential biomarkers, there are certain criteria that must be fulfilled: they should be quantitatively altered in AMD; they should demonstrate organ- and cell-specific expression patterns; they should be easily accessible; and they should be able to demonstrate sufficiently high stability through storage and testing conditions.

To attain the first goal, stringent selection and categorization of the phenotypes of patients is imperative. Ascertaining a true control population without AMD and other retinal disease would be of utmost importance. Additionally, it is vital to establish correct phenotyping of AMD patients without any evidence of other retinal disease versus mimicking diseases such as central areolar choroidal dystrophy [157], Malattia leventinese [158], and others. Secondly, as peripheral serum or plasma are the most likely screening modalities, it will be imperative to rule out any concurrent systemic processes, as mRNA transcripts can be altered in states of inflammation [159,160], oxidative stress [161], and cell death [162,163], which can occur in a variety of different acute and chronic diseases. 

Once a sufficiently reliable repository of AMD patients is established, the remaining step would be much simpler and would depend on the goal of the experiment. Charting the clinical course of patients with wet AMD versus those without wet AMD would require retrospective samples related to the specific patients. Therefore, obtaining samples longitudinally would be essential. This approach may highlight unknown avenues of biologic dysregulation important in the disease process that may advance our understanding of the disease and allow for the discovery of new potential therapeutic targets.

## 3. ncRNAs as Potential Therapeutic Targets

### 3.1. Benefits for Using ncRNAs as Therapeutic Targets

One major advantage of RNA based therapy is the ease of development. Unlike small molecules and antibodies, appropriate oligonucleotides can be designed to target specific mRNAs, so long as their transcripts are readily available. Additionally, ncRNAs offer the ability to target traditionally undruggable targets [164,165]. This includes complex proteins for which it may be difficult to block all key functions, complex protein-protein interactions, and proteins with high homology making it nearly impossible to achieve selective repression of a single protein [164,165]. 

**Figure 1:**
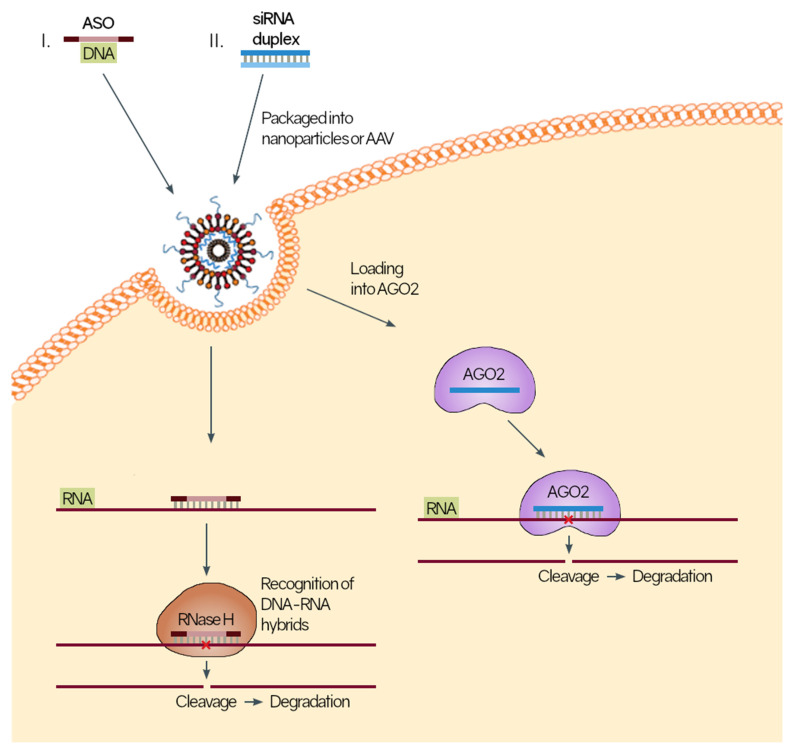
Mechanisms of mRNA transcript regulation. I. Anti-sense oligonucleotides (ASO) or II. Small interfering RNA (siRNA) duplexes are packaged into nanoparticles or adeno-associated viruses (AAV) that allow them to pass through the lipid bilayer. Once inside, ASOs can bind to the complementary messenger RNA (mRNA) strands. These DNA-RNA hybrids are recognized by RNAse H, leading to cleavage and degradation of the transcript, ×, indicates where RNAse H is binding. siRNA duplexes loaded into Argonaute 2 (AGO2) to form an RNA-induced silencing complex that can bind to complementary mRNA strands, leading to cleavage and degradation of the transcript. Both pathways lead to significant downregulation of gene expression.

The most common modalities of therapeutic application have been through controlling mRNA expression with antisense oligonucleotides (ASOs) and duplex RNAs (Figure 1). ASOs function by forming DNA:RNA hybrids which recruit RNase H, leading to degradation of the complementary mRNA strand. Duplex RNAs work directly by the intrinsic RNA interference system and actively represses and degrades mRNA transcripts [166]. Both modalities result in a repression of the target protein, in which traditional molecules cannot inhibit both the intracellular and extracellular effects of the targeted protein. This effect can theoretically be bolstered by complementing the ncRNA therapeutic with traditional therapeutics as they work through different mechanisms. Similar to monoclonal antibodies, an additional benefit of RNA-based therapeutics is the intrinsically high specificity in targeting only complementary strands of DNA with relatively high fidelity. The modern design of ASOs consists of a central DNA region flanked by chemically modified nucleotides. This has allowed for improved complementary targeting, as well as increased resistance to nuclease cleavage [167,168]. This can substantially increase half-lives and reduce the clinical burden of repeated dosing. Currently, the treatment for wet AMD relies on antibodies which have a relatively fixed half-life in ocular fluids, and often requires monthly dosing of intravitreal anti-VEGFs. This concept has been applied in the cardiovascular literature with the RNAi of PCSK9, where the efficacy in reducing LDL was similar to that of PCSK9 antibodies but only required bi-annual dosing [169].

### 3.2. Challenges for Using ncRNAs as Therapeutic Targets

RNA molecules are incredibly versatile tools that can carry information in a linear sequence of nucleotides to target specific sequences but can also become incredibly complex as they have a secondary and tertiary structure that can lead to many off target effects [170]. The major challenges that need to be overcome to develop an effective ncRNA therapeutic include determining the specific pathologic process to target in AMD, attaining sufficient delivery and stability in vivo, attaining high specificity of ncRNA for their molecular targets, and attaining high specificity of targeting the intended cell type to minimize the adverse effects of the therapeutic itself or the drug delivery system utilized. 

A major challenge when designing studies for ncRNA therapeutics is the limitation of the traditional cell line, animal model, and human study paradigm. It is evident that most of the animal and cell models of AMD will not capture the entire progressive nature of the disease. However, they have still been instrumental in the development and screening of potential therapeutic targets. ncRNAs not only demonstrate species to species variation in their function, but also tissue to tissue variation which may be disrupted during the immortalization process of cell lines. Therefore, even if a particular miR with known efficacy in a human cell line appears promising, the results may be difficult to translate into real human tissue and small proof of concept studies in humans will ultimately be the deciding factor for further validation studies. 

Although RNA is intrinsically designed to be a transient molecule with a stability that pales in comparison to DNA, multiple advances in RNA technology have helped with improvement of delivery and stability in vivo. Broadly speaking, RNA delivery can be performed using viral and non-viral vectors. Some of the initial models have been adapted from DNA delivery models such as engineered adeno-associated viruses (AAV) [171]. Utilization of nanoparticles is a newly evolving field, whereby unique compounds encapsulate the RNA. The first generation of these particles involved cationic polymers to electrostatically condense the negatively charged RNA and protect them from degradation [172]. However, lipid-like materials have proven to have additional benefits in transferring genetic information by improved endosomal escape [173], reducing systemic toxicity [174] and enhancing nanoparticle stability and delivery [175]. 

To enhance in vivo stability, chemical modifications to the RNA structure have been used to enhance resistance to endogenous RNAses [176] and render them less likely to trigger immune reactions [177]. The degree to which these modifications affect their intracellular interactions is dependent on the mechanism of action of the RNA, with those relying on the endogenous RNA-induced slicing complex (RISC), allowing heavy modification while retaining high levels of biologic function [178]. However, as we move towards other longer forms of ncRNA therapeutics, it is important to be cautious in relying on these methods, as studies have shown that large RNA compounds, such as mRNA, are highly sensitive to the currently utilized modified bases and thus must be approached with caution when developing lncRNA therapeutics [179,180]. 

In terms of specific targeting to ophthalmic tissue, ncRNA therapeutics have two potential avenues, both which have demonstrated high levels of success in prior studies. Owing to the unique anatomic compartmentalization of the eye, targeting the subretinal space via surgical procedures is indeed possible as demonstrated by prior and ongoing ocular gene therapy trials [181]. Additionally, trials in cardiovascular medicine have demonstrated the ability to target specific tissue, such as the liver, through modification of surface lipid nanoparticular delivery vectors delivered systemically [182,183]. Similarly, another study has demonstrated the ability of modified lipid nanoparticles to target the lung epithelium directly [184]. It is conceivable that development of a similarly selective nanoparticle that targets the RPE or choroid cells would be possible as our understanding of lipid nanoparticles advances.

### 3.3. Guidance in Using ncRNAs as Therapeutics

In ophthalmology, there are currently no clinical trials using ncRNAs as therapeutics. However, we can take heed of the lessons learned from other medical fields to optimize the potential for utilization of ncRNAs as therapeutics in AMD. 

The most challenging aspect of drug design involves selection of the modality to target and design the oligonucleotide sequence itself. As mentioned above, ASOs and duplex RNAs are the most well studied mechanisms of RNA modulation. However, the most challenging aspect of drug design has been controlling off-target effects within cells [185,186,187]. Although expected target mRNA sequences may often be affected, these molecules are complex and can lead to off-target effects through multiple mechanisms including those due to secondary and tertiary conformational effects [188,189]. To account for these effects, diligent control experiments are often performed with the following suggestions outlined by Myers and Stein [190,191]. Typically, by using two or three compounds that target the same oligonucleotide sequence, one can be confident the resultant phenotype is purely due to the on-target effects with minimal changes in unintended targets [190,191]. Additionally, adequately screening with the database of genomic DNA and transcript sequences can help identify potential drug candidates with a high likelihood of off-target effects. Due to the nature of small RNA motif interactions, it would also be important to perform control studies using scrambled (groups of bases swapped within the controls) as well as mismatched (mismatched bases relative to the target) sequences. These should also be done using compounds with groups of bases in the same order, as small motifs as benign as CpG dinucleotides have been demonstrated to stimulate the immune system independent of the flanking sequences [192]. 

The second most important consideration is the appropriate selection of tissue. As emphasized throughout this review, due to species-to-species variation, performing proof of principle animal studies will be difficult, and only feasible in those where an equivalent analog of the targeting human equivalent is proven not only at the species level, but also at the level of the targeted cell equivalent. If the aforementioned control studies were reproducible with both human and animal RPE cell lines, one would have greater confidence that animal studies would be more representative of success in a human clinical trial. After this stage, one of the major benefits of RNA is that because the delivery systems have been well validated in previous studies, either AAV or nanoparticles need only be modified or injected in a manner similar to previous studies. The major lag that occurred in the field between the initial discovery of RNA inhibition and the development of therapeutics is largely attributed to the work required to improve RNA stability and delivery as covered in the previous section. With the groundwork already laid out, now is the opportunity for the field of ophthalmology to look towards ncRNA targeted therapeutics as the next innovation in AMD care.

## 4. Conclusions

AMD is a complex disease with a major medical and economic impact, and understanding of the disease’s mechanisms continues to evolve. This review discusses the role that ncRNA may play in expanding our understanding and treatment of AMD. Diagnostically, ncRNA have been demonstrated in other medical fields to confer benefits such as earlier detection, prognostication, and biomarker-guided therapy. Clinical trials with ncRNAs as therapeutic targets have shown success in other fields and may serve as a new avenue of investigation in AMD. For future clinical application, appropriate sources of disease-affected tissue coupled with patient populations in which AMD and normals are well characterized are critical for identifying ncRNA biomarkers, but also in identifying targets for future therapeutics.

## Figures and Tables

**Table 1:** Studies Evaluating Micro RNAs as Biomarkers for AMD.

Findings	Patients	Tissue	AMD Types	Validation Method	Age	Methodology	Reference
Increased in dry and wet: miR-Let-7, miR-301-3p, miR-424-5p, miR-438, miR-661, miR-889, miR-3121, miR-4258	300 AMD (150 dry and 150 wet) 200 controls	Serum	Dry and Wet	OCT and IVFA	68 for AMD, 67 for controls	qRT-PCR	Szemraj [47]
Increased in wet: miR-301-3p, miR-361-5p, miR-424a-5p	129 AMD, 147 controls	Plasma	Wet	Fundus exam	75–80 for AMD, 73–78 for controls	qRT-PCR	Grassmann [48]
Increased in wet: miR-146a-5p Decreased in wet: miR-106b-5p, miR-152-3p	13 AMD, 13 control	Plasma and vitreous	Wet	OCT	82 for AMD, 67 for controls	Microarray and qRT-PCR	Menard [49]
Increased in wet: miR-Let-7c, miR-17-5p, miR-20a-5p, miR-24-3p, miR-26b-5p, miR-27b-3p, miR-29a-3p, miR-106a-5p, miR-139-3p, miR-212–3p, miR-223-3p, miR-324-3p, miR-324-5p, miR-532-3p, miR-744-5p, and Decreased in wet: miR-21-5p, miR-25-3p, miR-140-3p, miR-146b-5p, miR-192-5p, miR-335-5p, miR-342-3p, miR-374a-5p, miR-410, miR-574-3p, and miR-660-5p	33 AMD, 31 controls	Plasma	Wet	OCT and IVFA	72 for AMD, 63 for controls	qRT-PCR	Ertekin [50]
Increased in dry and wet: miR-27a-3p, miR-29b-3p, miR-195-5p	132 AMD, 146 Control	Whole blood	Dry and Wet	Fundus photograph, OCT, IVFA	58 AMD, 55 Control	Microarray and qRT-PCR	Ren [38]
Increased in wet: miR-486-5p, miR-626 Lower in wet: miR-885-5p	70 AMD, 50 controls	Serum	Wet	Fundus exam	71 AMD, 70 Control	qRT-PCR	Elbay [51]
Increased in wet: miR-9, miR-23a, miR-27a, miR-34a, miR-126, miR-146a Decreased in wet: miR-155	11 AMD, 11 controls	Serum	Wet	IVFA	70 AMD, 70 Control	qRT-PCR	Romano [52]
Increased in dry and wet: miR-19a, miR-126, miR-410	80 AMD (40 wet and 40 dry), 40 controls	Serum	Dry and Wet	Fundus Exam	55+ for AMD and controls	qRT-PCR	ElShelmani [42]
Increased in dry: miR-23a3p, miR-126-3p, miR-126-5p, miR-146a Increased in wet: miR-23a3p, miR-30b, miR-191-5p, and miR-223-3p Decreased in dry: miR-16-5p, miR-17-3p Decreased in wet: miR-16-5p, miR-17-3p, miR-150-5p, and miR-155-5p	354 AMD, 121 controls	Peripheral blood cells	Dry and Wet	OCT	73 Dry, 74 Wet, 73 Control	qRT-PCR	Litwinska [53]

## Data Availability

Not applicable.

## Abbreviations

AMD	age-related macular degeneration
RPE	retinal pigment epithelium
AREDS	Age-Related Eye Disease Study
GA	geographic atrophy
MNV	macular neovascularization
FAF	fundus autofluorescence imaging
SD-OCT	spectral domain optical coherence tomography
OCTA	optical coherence tomography angiography
IVFA	intravenous fluorescein angiography
VEGF	vascular endothelial growth factor
ncRNAs	non-coding RNAs
mRNA	messenger RNA
miRNAs	micro RNAs
miRs	micro RNAs
lncRNAs	long non-coding RNAs
piRNAs	piwi interacting RNAs
circRNAs	circular RNAs
siRNAs	small interfering RNAs
ASO	anti-sense oligonucleotide
AGO2	argonaute 2
RISC	RNA-induced silencing complex
qRT-PCR	quantitative reverse transcription polymerase chain reaction
AAV	adeno-associated virus
